# “Do You Think It’s Dementia?” Could This Question Transform How We Deliver Dementia Diagnoses?

**DOI:** 10.1192/bjo.2026.11618

**Published:** 2026-06-30

**Authors:** Su Yi Mon, Simon Vann Jones, Allison O'Kelly

**Affiliations:** Cornwall Partnership NHS Foundation Trust, Cornwall, United Kingdom

## Abstract

**Aims:**

**Background:**

In 2021, in order to optimise the quality of informant questionnaires, the local memory service introduced the Brief Informant Questionnaire (BIQ), consisting of five short prompts that allow family members or carers to share their observations about memory changes, daily functioning, behaviour, and any potential risks such as safety concerns. As part of this, we asked the simple binary question “Do you think it is dementia”.

**Aim:**

- To evaluate the accuracy of a single item on the BIQ by comparing the informant’s response with the clinical diagnosis following comprehensive assessment.

**Methods:**

- Data was collected from the 50 randomly selected patients who attended the local memory clinic from 2022 to 2024. 5 informants did not provide the response to the question “Do you think it is dementia?” and they were not included. As such, 45 cases were included in the final analysis.
- In scenario 1, only definite “Yes” and “No” responses were used to evaluate the diagnostic performance.
- In scenario 2, “Not sure” responses were combined with “Yes” responses.

**Results:**

**Scenario 1:**

Patient had dementia and informant agreed: 28/29 Sensitivity: 96.5%

Patient did not have dementia and informant agreed: 5/5 Specificity: 100%

Positive Predictive Value: 28/28=100%

Negative Predictive Value: 5/6=83.3%

**Scenario 2:**

Patient had dementia and informant agreed or was not sure: Sensitivity: 34/35=97.1%

Patient did not have dementia and informant agreed or was not sure: Specificity: 5/10=50%

Positive Predictive Value: 34/39=87.1%

Negative Predictive Value: 5/6=83.3%

**Conclusion:**

The BIQ demonstrates very high sensitivity in both analysis scenarios (96.5% and 97.1%) and a strong positive predictive value (100% and 87.1%), indicating that an affirmative response was highly indicative of a true dementia diagnosis.

When “not sure” responses were grouped with “yes,” the specificity decreased, reflecting reduced ability to identify non-dementia cases.

Although the number of negative cases was small, the findings suggest that the BIQ performs reliably within the current assessment pathway and is clinically useful.

Completing this questionnaire prior to the initial assessment helps ensure that relevant information is available from the start, improves the quality and efficiency of the assessment process, and supports more person-centred assessments and care planning.

Overall, these findings provide evidence to support the use of this single item from the BIQ across wider memory services as an aid to identifying possible dementia.

